# Calcinose tumorale chez un hémodialysé

**DOI:** 10.11604/pamj.2016.23.155.8335

**Published:** 2016-03-31

**Authors:** Manel Jellouli, Tahar Gargah

**Affiliations:** 1 Service de Pédiatrie, Hôpital Charles Nicolle, Tunis, Tunisie

**Keywords:** Calcinose tumorale, dialyse, insuffisance rénale, Tumoral calcinosis, dialysis, kidneys failure

## Image en médecine

La calcinose tumorale est une affection bénigne rare, qui se caractérise par le dépôt de matériel calcique dans les tissus mous extra-articulaires prenant une forme tumorale. Elle peut être primitive ou secondaire à une insuffisance rénale chronique. Nous rapportons un nouveau cas de calcinose tumorale chez un jeune homme âgé de 27 ans. Ce patient est en insuffisance rénale terminale secondaire à une uropathie malformative depuis 15 ans, traité initialement par dialyse péritonéale puis par hémodialyse. Le patient présentait depuis 5 mois une tuméfaction progressive de la hanche le gênant à la marche. L'examen trouvait une infiltration de consistance pierreuse. Il n'y avait pas de lésion cutanée anormale. La radiographie du bassin mettait en évidence d'importants dépôts calciques diffus dans le tissu souscutané. La scintigraphie osseuse montre une hyperfixation en regard de la hanche. L'imagerie par résonnance magnétique trouvait une calcinose tumorale de la face postérieure de la racine de la cuisse mesurant 14x13x12 cm bien limitée hétérogène avec fine prise de contraste périphérique et des septas, situé au niveau du muscle grand fessier, grand adducteur et prenant la partie proximale du tendon conjoint du biceps fémoral et semi tendineux. Cette masse arrive au contact du nerf sciatique sans engagement du derrier. La biologie trouvait une calcémie à 2,33 mmol/L, phosphorémie à 3,1 mmol/L, parathyroide hormone (PTH) à 770 ng/L et créatinine à 977 mol/L. Une indication opératoire était proposée mais refusé par le patient.

**Figure 1:**
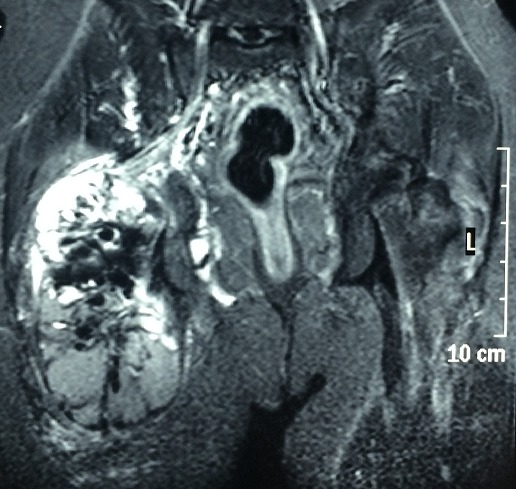
l'imagerie par résonnance magnétique montrant une calcinose tumorale de la face postérieure de la racine de la cuisse bien limitée hétérogène situé au niveau du muscle grand fessier, grand adducteur sans engainement du nerf sciatique

